# A Novel Workflow to Create a Checkpoint Inhibitor Pneumonitis Patient Registry

**DOI:** 10.7759/cureus.34683

**Published:** 2023-02-06

**Authors:** Andrew Faucheux, Eric Olson, Jeffrey Lantz, Nathan Roberts, Vanya Aggarwal, Indra Newman, Janardhana Ponnatapura, Thomas Lycan

**Affiliations:** 1 Internal Medicine, Wake Forest University School of Medicine, Winston-Salem, USA; 2 Hematology and Oncology, Wake Forest University School of Medicine, Winston-Salem, USA; 3 Hematology and Oncology, University of Virginia School of Medicine, Charlottesville, USA; 4 Hematology and Oncology, Georgetown University Medical Center, Washington, DC, USA; 5 Wake Forest Clinical and Translational Science Institute, Wake Forest University School of Medicine, Winston-Salem, USA; 6 Radiology, Wake Forest University School of Medicine, Winston-Salem, USA

**Keywords:** immune checkpoint inhibitor, checkpoint inhibitor pneumonitis, registries, mimics, toxicity, immune-related adverse events, immunotherapy

## Abstract

Background

Despite being a groundbreaking cancer therapy, immune checkpoint inhibitors (ICI) can lead to potentially life-threatening toxicity with checkpoint inhibitor pneumonitis (CIP). While treatable, it is easy for clinicians to miss the symptoms of CIP, which can lead to a delay in diagnosis and worsening respiratory function. There is no consensus approach to systematically identifying patients at risk of developing CIP. Thus, we sought to create a workflow that could inform patient selection for ICI therapy based on previously reported risk factors for CIP development.

Materials and methods

We retrospectively identified 250 patients with lung cancer treated with at least one dose of an ICI over 20 months. Data were collected on comorbidities, cancer type and stage, performance status, ICI cycles, biomarkers, prior curative treatment, diagnostic evaluation, antibiotics, steroids, progression, and survival. A single-blinded radiologist characterized radiographic patterns of suspected CIP cases.

Results

Among 97 patients who received steroids while admitted to the hospital, 12 (6%) had at least one sign or symptom suggestive of CIP. Chronic obstructive pulmonary disease and non-small cell lung cancer subtypes correlated with suspicion of having CIP. CIP was confirmed in five patients (42%) and ruled out (mimics) in seven (58%). Median times until symptoms were 17 months and one month for confirmed and mimic cases, respectively. The median time to confirm or exclude CIP was 5 ± 4 days. Most suspected cases underwent thoracic imaging, blood cultures, and empiric antibiotics. Radiographic patterns in suspected cases included ground glass opacities, organizing pneumonia, acute interstitial pneumonia/acute respiratory distress syndrome, bronchiolitis, radiation recall pneumonitis, hypersensitivity pneumonitis, and post-radiation fibrotic changes.

Conclusions

CIP mimics are common in clinical practice; therefore, it is reasonable to empirically treat suspected cases with shorter courses of steroids until diagnostic clarity is achieved. This proof-of-concept study demonstrates that this novel workflow can identify the true incidence of CIP, inform treatment decisions, and lead to the development of implementation studies to improve patient care directly.

## Introduction

Although immune checkpoint inhibitors (ICI) improve lung cancer outcomes, they sometimes cause severe and even fatal immune checkpoint inhibitor pneumonitis (CIP) [1,2]. It is often difficult for clinicians to make a timely CIP diagnosis, as there are no validated criteria or diagnostic algorithms to discern CIP from the many other causes of acute respiratory failure in patients with lung cancer [3,4]. The uncertainty of confirming the diagnosis of CIP is reflected by the wide variability in the reported incidence rates of CIP [5]. In clinical trials, the incidence of CIP is approximately 3% for monotherapy targeting either programmed cell death protein-1 (PD-1) or its ligand (PD-L1) and 7% for dual inhibition of PD-1 and cytotoxic T-lymphocyte-associated protein 4 [6-8]. However, the “real-world” incidence of CIP is thought to be much higher than that seen in clinical trials and ranges somewhere between 3% and 21% [9-11]. There is a critical need to confirm the real-world incidence of CIP among patients who develop acute respiratory failure while being treated for lung cancer.

Patient registries have provided the majority of research on CIP because of the low incidence of immune-related adverse events among patients eligible for clinical trials. However, most of these registries do not have a systematic method for identifying patients with CIP, consisting of patients who were identified by clinical trial monitoring or referred by their provider to the registry for a CIP clinical diagnosis or characteristic radiographic pattern [12-14]. Professional organizations have prioritized the development of novel registries that systematically collect data on all patients receiving an ICI who develop acute respiratory failure, i.e., not only CIP but also other etiologies (CIP “mimics”) [4]. We aim to develop a workflow to identify cases and provide attribution of respiratory failure using routine medical documentation for a patient registry.

## Materials and methods

This single-center retrospective study collected demographic and clinical data from individual patient charts within the electronic medical record and entered them into a case report form within a novel registry. We included all patients who had (1) an International Classification of Diseases, Tenth Revision (ICD-10) code for "malignant neoplasm of bronchus and lung" (C34) and (2) administration of at least one dose of a Food and Drug Administration (FDA)-approved ICI. We limited our sample to patients who received their first dose of ICI between 6/1/2018 and 2/1/2020. We cut off data collection at the local onset of the coronavirus disease 2019 pandemic to avoid the confounding created by an infectious outbreak of a respiratory virus. We obtained demographic and clinical data starting from the date of the first administration of ICI until the last known follow-up. We collected toxicity outcomes for all adverse events that were either high grade (i.e., grade 3-5 or persistent grade 2) or severe. Toxicity outcomes included but were not limited to skin toxicity, colitis, hepatitis, pneumonitis, thyroid disease, adrenal or pituitary disease, nephritis, diabetes, and inflammatory arthritis. We defined severe toxicity as events that (1) required urgent intervention or hospitalization; (2) were prolonged despite symptomatic management; (3) led to delay of chemotherapy or ICI; or (4) had life-threatening consequences. We did not collect data on outcomes from toxicities that were solely an abnormal laboratory value, required no intervention, and met no criteria for severe toxicity. The registry was created and managed on our institution’s secure, web-based Research Electronic Data Capture tool [15]. The institutional review board approved the study protocol. Categorical variables are reported as frequencies (percentages with 95% CIs) and continuous variables as means (with standard deviations) or medians (with interquartile ranges and 95% CIs) according to distribution.

Development of a novel workflow to identify severe immune-related adverse events

**Figure 1:**
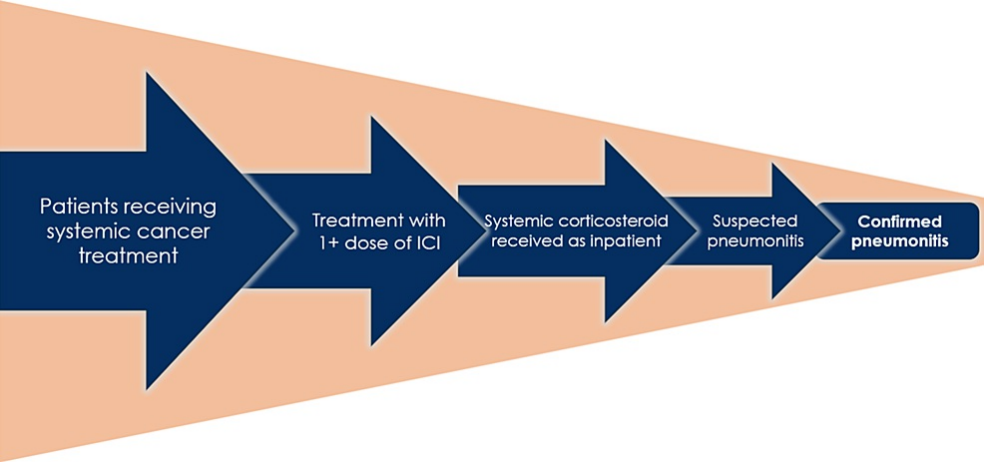
Novel workflow A deductive approach to identifying patients with confirmed pneumonitis immune-related adverse events. ICI: immune checkpoint inhibitor.

Similar to other institutions, our historical approach to immune-related adverse event (irAE) surveillance has been a registry of patients with pneumonitis whom a treating clinician referred to the research group. This approach has been insufficient because of the high volume of new patients and the subjective nature of the referrals, excluding possible or probable pneumonitis cases. Therefore, to develop a systematic approach to identify cases of suspected CIP rapidly, we sought to build an inventory of severe treatment-emergent adverse events. First, we identified patients who received at least one dose of a systemic steroid during an inpatient encounter. We then collected data on outcomes from all adverse events regardless of attribution (i.e., treatment-emergent) and not just those attributed to ICI (i.e., treatment-related). We defined cases of suspected pneumonitis by the presence of any one of the general clinical or radiographic criteria listed by professional guidelines [3]. Suspected cases were then categorized as either confirmed CIP or ruled out for CIP (i.e., CIP mimic). We defined a CIP mimic as an acute event that seemed less likely than not (i.e., < 50%) to be an irAE as opposed to another more plausible etiology, as demonstrated by (1) atypical presentation for an irAE, (2) resolution of symptoms by treating another etiology, or (3) no recurring symptoms after restarting ICI therapy. We categorized cases not meeting the above criteria as confirmed CIP (Figure 1). We defined time to diagnostic clarity as the time from the first presentation to a healthcare provider until there was either documentation that providers had confirmed the diagnosis or until providers discontinued any empiric treatments, whichever occurred first. A thoracic radiologist blinded to clinical documentation reviewed all imaging of suspected CIP cases and described their radiographic patterns. Our group’s historical method for CIP surveillance identified none of these 12 cases.

## Results

Application of the novel workflow to identify cases of suspected pneumonitis

We identified 250 patients with lung cancer treated with at least one dose of an ICI over 20 months. Among those patients, 97 (46%) received systemic steroids during hospitalization. Table 1 summarizes their demographic and clinical characteristics. The majority were over 60 years old, male, Caucasian, had concurrent chronic obstructive pulmonary disease (COPD), and had received at least one line of systemic cancer-directed therapy before ICI. Follow-up was 23 months, with progression in 54 patients (56%) at a median of 11 months and death in 67 patients (69%) at a median of 14 months. Among patients who received steroids while inpatient, 12 (6%) had at least one sign or symptom of a possible pneumonitis. COPD and the non-small cell lung cancer subtypes were more prevalent among patients suspected of having CIP. Of these 12 patients, CIP was ruled out (mimics) in seven and confirmed in five patients. Patients with CIP mimics were more likely to have an early stage of lung cancer (i.e., stage I-II) and a cancer treatment history of multiple modalities. All patients with confirmed CIP cases were at least grade 3 irAE or higher, and one death (grade 5) was due to CIP. In terms of follow-up, death occurred in six participants (6/7) with CIP mimics and two participants (2/5) with confirmed CIP. The causes of death among patients with CIP mimics were primarily due to infectious etiologies (5/6). The causes of death among patients with confirmed CIP were due to CIP (1/2) or infection (1/2). Our group’s historical method of CIP surveillance identified none of these 12 cases during the study period.

**Table 1:** Baseline demographic and clinical characteristics (n = 97) * One patient was exposed to asbestos, and another was exposed to “hazardous dust/chemicals”. ^†^ One patient had previous occupational exposure to asbestos, diesel fumes, and coal. ^‡^ One patient had metastatic left tonsillar squamous cell carcinoma, and another had localized squamous cell carcinoma of the forehead. ^§^ One patient had prior localized squamous cell carcinoma of the larynx. ** Three patients also had prior treatment with an immune checkpoint inhibitor (durvalumab followed by pembrolizumab; pembrolizumab followed by nivolumab; and pembrolizumab followed by durvalumab). CIP: checkpoint inhibitor pneumonitis; ICI: immune checkpoint inhibitor; COPD: chronic obstructive pulmonary disease; NSCLC: non-small cell lung cancer.

		CIP never suspected (n = 85)	CIP suspected, ruled out (n = 7)	CIP suspected, confirmed (n = 5)
Age in years at the start of ICI, mean (range)		67 (47 - 84)	67 (57 - 79)	64 (59 - 70)
Male gender, n (%)		50 (59%)	5 (71%)	3 (60%)
Race, n (%)				
	White or Caucasian	71 (84%)	5 (71%)	3 (60%)
	Black or African American	12 (14%)	2 (29%)	2 (40%)
	Hispanic	1 (1%)	0	0
	Pacific Islander	1 (1%)	0	0
Smoking status, n (%)				
	Never	7 (8%)	0	0
	Former	47 (55%)	5 (71%)	4 (80%)
	Current	31 (36%)	2 (29%)	1 (20%)
	Median pack years	40	52	47
Occupational exposure, n (%)		15 (18%)	2* (29%)	1^†^ (20%)
History of COPD/asthma, n (%)		45 (53%)	6 (86%)	4 (80%)
Autoimmune disease, n (%)		1 (1%)	0	0
Prior malignancy, n (%)		23 (27%)	2^‡^ (29%)	1^§^ (20%)
Lung cancer type/subtype, n (%)				
	NSCLC, non-squamous	46 (54%)	1 (14%)	4 (80%)
	NSCLC, squamous	25 (29%)	5 (71%)	1 (20%)
	Small cell lung cancer	14 (16%)	1 (14%)	0
Initial stage at diagnosis, n (%)				
	I	6 (7%)	0	2 (40%)
	II	5 (6%)	1 (14%)	1 (20%)
	III	20 (24%)	2 (29%)	0
	IV	54 (64%)	4 (57%)	2 (40%)
Treatment prior to ICI initiation, n (%)				
	Surgery (curative intent)	8 (9%)	3 (43%)	1 (20%)
	Radiation (curative intent)	23 (27%)	3 (43%)	2 (40%)
	Systemic therapy	37 (44%)	5 (71%)	3 (60%)
	No prior therapy	17 (20%)	2 (29%)	2 (40%)
IO treatment used, n (%)				
	Pembrolizumab**	53 (62%)	3 (43%)	2 (40%)
	Nivolumab**	24 (28%)	4 (57%)	3 (60%)
	Atezolizumab	4 (5%)	0	0
	Durvalumab**	2 (2%)	0	0
	Nivolumab + ipilimumab	2 (2%)	0	0

Use of the novel workflow to determine the attribution of respiratory failure

**Figure 2:**
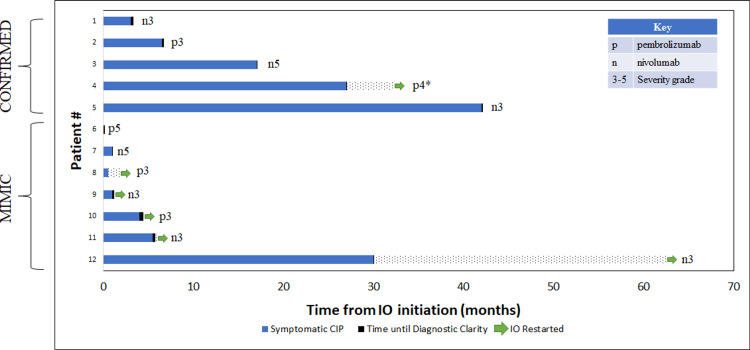
Confirmed and mimic cases Swimmer plot outlining months until symptomatic CIP (blue), diagnostic clarity (black), and IO restarted (green arrow). Time to diagnostic clarity indicates time spent to differentiate mimics from confirmed cases. * Resumption of IO resulted in the recurrence of respiratory symptoms. Key shows IO used and suspected initial severity. CIP: checkpoint inhibitor pneumonitis; IO: immune checkpoint inhibitor.

The majority of suspected cases underwent chest X-rays, chest CTs, and blood cultures, and received empiric antibiotics. The majority of ruled-out cases (4/7) and all confirmed cases (5/5) had a chest CT. All confirmed cases and five of seven mimics received more than one administration of high-dose corticosteroids (defined as at least 0.5 mg/kg/day prednisone equivalent). The median duration of high-dose steroid treatment for confirmed and mimic cases was eight days and one day, respectively. Common radiographic patterns identified in the ruled-out cases included bronchiolitis (2/7), organizing pneumonia (1/7), ground-glass opacities (1/7), and acute interstitial pneumonia (AIP)/acute respiratory distress syndrome (ARDS) pattern (1/7). Two ruled-out cases demonstrated no discernible features to suggest CIP. Patterns demonstrated in the confirmed cases were organizing pneumonia (2/5), radiation recall pneumonitis (1/5), hypersensitivity pneumonitis (1/5), AIP-ARDS (1/5), ground-glass opacities (2/5), and post-radiation fibrotic changes (2/5). Multiple images from confirmed cases revealed concomitant patterns, and a distinctive pattern was not identified in two out of five cases. The majority of confirmed cases (4/5) had previously received radiation therapy with curative intent. Three of five confirmed cases revealed signs of post-radiation changes on CT chest imaging, and one revealed a radiation recall pneumonitis pattern. Our results are consistent with previous studies, which report organizing pneumonia and ground-glass opacities as the most common patterns present in CIP. Time to diagnostic clarity (TDC), defined as the time from symptom onset until confirming or ruling out CIP, occurred after a median of five days (range: two to seven days) for confirmed CIP cases and after a median of four days (range: one to 13 days) for CIP mimics (Figure 2).

## Discussion

Interpretation of data

Our study demonstrates the feasibility of systematically collecting real-world toxicity data on lung cancer patients with suspected CIP and provides early data on the proportion of CIP mimics. To our knowledge, it is the first retrospective dataset to systematically include all patients who were suspected at any point to have CIP, including those who later had it ruled out on subsequent workup. We found a high prevalence of CIP mimics relative to the number of confirmed cases and a delayed time to diagnosis (greater than two days) in all cases, even with the availability of advanced imaging.

Importantly, this methodological approach allows direct comparison between CIP and other diagnoses with similar presentations. We found that some measures differed substantially between confirmed cases and mimics and may help to confirm or rule out CIP. In particular, mimics of CIP tended to occur much earlier in the ICI treatment course (one month), while confirmed cases of CIP tended to happen later. This delayed onset is consistent with prior literature that reported a median time to pneumonitis development of two to three months [9,16,17]. These findings, combined with our data, suggest that respiratory symptoms early in the course of ICI treatment (especially under one month) are more likely to result from another etiology. A possible mechanism for this is the late emergence of pathogenic T cells from a larger number of clonotypes following checkpoint blockade [18].

We noted an overlap in radiographic patterns in confirmed cases and mimics, suggesting that imaging is limited in its ability to rule out mimics. Of confirmed cases, the two most common patterns identified were organizing pneumonia and ground glass opacities (both seen in 40% of confirmed cases), which is consistent with current literature [19-21]. Interestingly, confirmed cases were more likely to have at least two concomitant patterns, with the significance of this observation being unknown. We found that patients with either a ground glass or an AIP-ARDS pattern seemed to have shorter overall survival, which was consistent among both confirmed and ruled-out cases. These findings are consistent with previously reported datasets, which also found a worse prognosis for CIP that had these radiographic patterns [22]. It is not surprising that our imaging studies between the two groups were largely indistinguishable, as characterized radiographic patterns for irAEs have been reported to be indistinguishable from other non-irAE pathologies [19,23]. Our results reiterate the importance of using imaging predominantly as a screening tool in the workup of CIP. As there are no pathognomonic radiographic features for CIP, imaging should be used with additional diagnostic workup.

Demographic and clinical factors were not associated with the incidence of CIP mimics. The majority of patients with confirmed CIP had received prior radiation therapy (RT) for either curative or palliative intent and also had post-radiation CT changes on imaging. Previous studies have failed to show any statistically significant association between prior RT and the development of CIP; however, these studies consistently report a higher incidence of pneumonitis after RT [11,24,25]. Although no causal relationship has been demonstrated between previous RT and the development of CIP, there is an associated incidence found in confirmed cases that should not be disregarded. Furthermore, the majority of suspected cases received prior chemotherapy with curative intent. Prior chemotherapy, therefore, does not seem to play a role in the development of CIP, nor does it aid in differentiating mimics. Notably, our study showed a trend of increased confirmed CIP cases associated with earlier stages of cancer at initial diagnosis. One explanation for these findings is that higher-stage lung cancers correlate with higher pre-existing pulmonary pathology and an increased propensity for alternative diagnoses, namely, bacterial pneumonia. Suresh et al. [9] appreciated similar results, postulating that lower-stage cancers may contribute more toward "priming" the lung for CIP after ICI treatment.

Limitations

Our study has several limitations, most notably a small sample size. Second, the study's retrospective nature may have resulted in information and selection bias. Third, the lack of reported data in the medical record limited analysis, particularly with respect to performance scores and PD-L1 expression. In terms of external validity, the recent development of extended dosing intervals for ICI may inherently reduce the frequency of unnecessary dose interruptions; however, our findings remain relevant for managing corticosteroids and other supportive care measures.

External validity

The lack of diagnostic clarity for CIP likely contributes to the discrepancy between the incidence of CIP in clinical trials versus real-world datasets. A meta-analysis that investigated CIP incidence among clinical trial participants with melanoma, NSCLC, or renal cell carcinoma reported an overall incidence of 2.7% during PD-1 inhibitor therapy and an incidence of 0.8% for grades three and higher [6]. Real-world clinical data have reported a significantly higher incidence, ranging from 3% to 21% for all grades and 3% to 4% for grades three and higher [9-11]. In our cohort, the incidence of confirmed cases of high-grade CIP was 5.2%, which was consistent with prior real-world datasets but higher than clinical trial datasets. Interestingly, the incidence of suspected CIP cases and CIP mimics was 12.4% and 7.2%, respectively. The incidence of suspected CIP reported in our study is closer to the real-world incidence of 3-21% reported in prior studies, which may suggest an over-reporting of CIP cases due to the inclusion of CIP mimics. Our data suggest that mimics are common in clinical practice and should be taken into account during the diagnostic workup and treatment of CIP.

Time to diagnostic clarity is an important measure that can prevent prolonged steroid administration and avoid unnecessary delay or discontinuation of ICI therapy. Unnecessary administration of steroids puts patients at risk for infections [26,27], hyperglycemia, and potentially decreased efficacy of ICI therapy [28,29]. These effects are more likely given long tapering recommendations (four to six weeks) for confirmed CIP and should be spared once it has been ruled out in cases of CIP mimics. Furthermore, CIP treatment guidelines recommend permanently discontinuing ICI therapy for grades 3-4 toxicities and potentially initiating additional immunosuppressives such as infliximab [16]. Notably, we found that the average and median time to diagnostic clarity in suspected cases of high-grade pneumonitis was 5.25 days and 4.5 days, respectively. Given this relatively short period, we believe it is reasonable to initiate corticosteroids for patients with suspicious presentations and later discontinue if CIP is ruled out. This aspect of the diagnostic workup is paramount, as it prevents adverse effects of prolonged steroid administration, potential permanent discontinuation of ICI therapy in high-grade CIP, and initiation of immunosuppressive agents.

## Conclusions

Using routine clinical documentation, this novel workflow systematically identified a cohort of patients for a CIP registry. None of these patients had been identified by our institution’s pneumonitis research group using the methodology (i.e., clinician referral) used by pharmacovigilance databases and the majority of CIP studies. By including patients who were suspected at any point to have possible CIP, it was able to overcome the difficulty of confirming a CIP diagnosis. This dataset demonstrates the feasibility of using this approach to study the individual diagnostic tests used to confirm or rule out CIP. For example, we found CIP-associated radiographic patterns in most patients with a CIP mimic. When used for a larger dataset, this workflow will allow for the development of positive predictive values for the different tests to diagnose CIP -- critical information for clinical guidelines. As previously prioritized by the American Thoracic Society, this novel workflow successfully identified cases of CIP mimics in addition to confirmed cases. This workflow is the first step toward defining the incidence rate of CIP, which can then inform treatment decisions and design implementation studies to improve patient care directly.
